# Learning from nature: the use of non-model species to identify novel acclimations to flooding stress

**DOI:** 10.1093/aobpla/plu016

**Published:** 2014-03-31

**Authors:** L. A. C. J. Voesenek, H. van Veen, R. Sasidharan

**Affiliations:** Institute of Environmental Biology, Utrecht University, Padualaan 8, 3584 CH Utrecht, The Netherlands

**Keywords:** Abscisic acid, climate change, ethylene, flooding, learning from nature, *Rorippa*, *Rumex*, waterlogging.

## Abstract

Excess water in the form of waterlogged soil or deeper submergence (generically termed ‘flooding’) influences plant growth, survival and species distribution in many natural ecosystems. It also has a negative impact on crop growth and yield since many agricultural species are flooding intolerant. The often devastating effect of flooding on plant performance is related to its interference with gas exchange between the plant and its environment. This results in energy deficiency and carbohydrate starvation. In the near future, flooding frequency is expected to increase due to global climate change and the human population is expected to increase to ∼9 billion people by 2050. The need for increased agricultural productivity is self-evident and this will require a better mechanistic understanding of the interaction between plants and abiotic stresses such as flooding. We argue that, in seeking this understanding, we should not restrict the research to model species such as rice (*Oryza sativa*) and Arabidopsis (*Arabidopsis thaliana*). This is because some stress-tolerance mechanisms are not found in these species. Examples are given of how flooding tolerance is achieved by non-model species of *Rumex* and *Rorippa* that have evolved to cope with flooding in natural environments. These findings will add usefully to the spread of resources available to plant breeding programmes aimed at improving flooding tolerance in crop plants.

## Abstract (precis)

Floods have a severe impact on plant performance. In general, crops are flood intolerant and are at an increased risk to flooding events due to global climate change. It is a grand challenge to improve agricultural production to feed the increasing human world population despite the increase of flooding stress. Mechanistic understanding of flooding tolerance is mainly achieved with model species such as *Oryza sativa* and *Arabidopsis thaliana*. However, wild plants from flood-prone environments have evolved in frequently flooded environments and therefore possess unique traits that facilitate growth and reproduction during and after flooding stress. Flooding research with these non-model wild plants might help us to identify novel adaptive traits that can be applied to improve flooding tolerance of crops.

## Introduction

Flooding in the form of waterlogged soil or deeper submergence of plants occurs in many natural ecosystems such as lakesides, river floodplains and (coastal) marshes. These flooding events vary in seasonal timing, duration, depth and frequency (Colmer and Voesenek 2009). This variation in flooding regime in combination with the variability of flooding tolerance between plant species determines patterns of plant distribution (Silvertown *et al.* 1999; Voesenek *et al.* 2004). Excess water can also decrease crop growth and yield since nearly all economically relevant crops are negatively affected by flooding (Voesenek and Bailey-Serres 2013).

Floods hamper plant performance because they interfere with normal gas exchange (oxygen (O_2_), carbon dioxide (CO_2_) and ethylene) and light availability needed for aerobic respiration and photosynthesis. These stress conditions deprive plants of essential carbohydrates and energy reserves and can, ultimately, kill the plant (Bailey-Serres and Voesenek 2008). However, plant species that inhabit permanently or temporarily flooded habitats use specific suites of traits to survive these harsh environments. Such species are, in general, characterized by high tissue porosities (aerenchyma) and restored shoot–atmosphere contact to facilitate internal gas diffusion (escape strategies) (Jackson and Armstrong 1999; Colmer 2003), and large carbohydrate reserves to fuel fermentation during phases of anaerobiosis when aerenchyma cannot conduct O_2_ effectively to inundated organs (e.g. when the whole plant is under water; Bucher *et al.* 1996). Other tolerance mechanisms include a dampening of energetically expensive processes such as growth (quiescence strategies; Bailey-Serres and Voesenek 2010) and modes to control cell damage by excessive amounts of reactive oxygen species (ROS) that develop during flooding and upon re-aeration when flood levels decline (Hunter *et al.* 1983; Monk *et al.* 1987; Blokhina *et al.* 2002; Santosa *et al.* 2007). Some species (e.g. *Phalaris arundinacea*, *Phragmites australis*, *Typha latifolia*) even possess gas films on their leaves that improve exchange of CO_2_ and O_2_ when leaves are submerged (Colmer and Pedersen 2007).

Despite this variation in adaptive traits in wild plant species, research on flood survival mechanisms has been mostly limited to flood-tolerant rice cultivars and the flood-intolerant model plant Arabidopsis*.* Over the past few years, work with these species has strongly accelerated the elucidation of molecular mechanisms that contribute to flooding tolerance. A notable example is the identification and functional characterization of the group VII ethylene response factor (ERF) transcription factor family in low O_2_ sensing, and in survival mechanisms related to escape and quiescence traits (Xu *et al.* 2006; Hattori *et al.* 2009; Gibbs *et al.* 2011; Licausi *et al.* 2011). However, these major discoveries do not explain why closely related wild plants and crops can vary enormously in flooding tolerance.

Wild plants from flood-prone areas have evolved in a selective environment that favours flood-adaptive traits. Furthermore, the resulting flood-adaptive traits have evolved in locations where neither rice nor Arabidopsis is found naturally. We can therefore assume that these wild species possess many as yet unknown flood tolerance genes and processes. The importance of this ‘Learning from Nature’ approach to understanding flooding tolerance mechanisms and harnessing this knowledge to develop crops with enhanced flood tolerance is illustrated and discussed here.

## *Rumex* and *Rorippa* Species: Variation in Flooding Tolerance and Molecular Survival Strategies

Two species from the genus *Rorippa* (*Ro. sylvestris* and *Ro. amphibia*) and two from the genus *Rumex* (*Ru. acetosa* and *Ru. palustris*) have been investigated extensively in recent studies of flooding tolerance and the underlying explanations. Compared to Arabidopsis and even rice, certain species from either genus are highly tolerant to complete submergence under dark conditions (Sasidharan *et al.* 2013). Under such demanding conditions, which typically prevent emergence of leaves, these four species can be ranked in the following order (from most to least tolerant): *Ro. sylvestris* > *Ro. amphibia* > *Ru. palustris* = *Ru. acetosa*. The corresponding average lethal time 50 (LT_50_) values under similar conditions were 32, 24, 20 and 20 days, respectively. When submerged, *Ro. sylvestris* and *Ru. acetosa* demonstrated strongly reduced elongation of the youngest leaf, suggesting that they employ a quiescence survival strategy. *Rorippa amphibia* and *Ru. palustris* show a mild to very strong elongation response of the youngest petiole, respectively (Akman *et al.* 2012; van Veen *et al.* 2013). This demonstrates that *Ru. palustris*, in particular, can escape submerged conditions via enhanced upward shoot elongation. Adaptive traits related to flooding such as enhanced shoot elongation and shoot porosity appear to act synergistically to enhance plant fitness (Colmer and Voesenek 2009). Consistent with this interpretation are observations that quiescent *Rumex* and *Rorippa* species have significantly lower petiole porosities than the escape species (Pierik *et al.* 2008; Akman *et al.* 2012).

Despite being highly tolerant, detailed investigations into the underlying molecular mechanisms in wild species such as *Rumex* and *Rorippa* have been hindered by the lack of suitable molecular genetic tools. However, recent studies have successfully applied global genome profiling techniques to characterize the molecular basis of flooding tolerance in the four plant species. While the close relatedness to Arabidopsis (both are genera of the Brassicaceaea) allowed the use of Arabidopsis ATH1 GeneChip arrays for both *Rorippa* species, deep sequencing techniques (RNAseq) were used for both *Rumex* species (Sasidharan *et al.* 2013; van Veen *et al.* 2013). In both studies plants were completely submerged, but in *Rorippa* the transcriptome profile was studied in whole root systems, whereas in *Rumex* only submerged young petiole tissue was used. Both systems are characterized by an accumulation of ethylene, but have significant differences in O_2_ concentrations (Voesenek and Sasidharan 2013). *Rorippa* plants submerged for 24 h have hypoxic to anoxic roots (Sasidharan *et al.* 2013); submerged *Rumex* petioles (4–10 h) have endogenous O_2_ levels between 17 and 21 % (van Veen *et al.* 2013).

The roots of both *Rorippa* species were characterized by a downregulation of genes involved in energy-demanding processes. Interestingly, glycolysis and fermentation genes were more strongly upregulated in the escaping *Ro. amphibia* compared with the quiescent *Ro. sylvestris* (Sasidharan *et al.* 2013), suggesting that this species has a stronger Pasteur effect (i.e. faster rates of sugar usage) and thus may produce more ATP per unit time. This would support cell maintenance and specifically in this species also help sustain the costly process of continued underwater shoot growth (Akman *et al.* 2012). Despite the presumed Pasteur effect, ATP levels declined in submerged *Ro. amphibia* (Sasidharan *et al.* 2013), indicating that maintenance and growth require more ATP than produced. In contrast, *Ro. sylvestris* employs a quiescence survival strategy during complete submergence. The prolonged survival of this species when completely under water is in line with its stable ATP content during submergence. This demonstrates that ATP production is in balance with the consumption required for maintenance (Sasidharan *et al.* 2013). Consistent with observations in rice, these results demonstrate that inhibition of energetically expensive elongation processes improves survival under conditions in which the water surface cannot be reached by means of upward shoot extension (Setter and Laureles 1996).

*Rumex acetosa* is characterized by reduced elongation growth upon submergence. That photosynthetically derived O_2_ and inward diffusion of O_2_ from the water layer can be sufficient to balance respiratory O_2_ use is indicated by high O_2_ levels found in submerged petioles (van Veen *et al.* 2013). A notable finding in *Ru. acetosa* was an induction of a trehalose phosphate phosphatase (TPP) during submergence. Trehalose phosphate phosphatase relieves trehalose-6-phosphate (T6P) inhibition on the heterotrimeric SnRK1 complex (Zhang *et al.* 2009), of which specific subunits are also transcriptionally activated in submerged *R. acetosa*. SnRK1 induces a variety of major catabolic pathways such as cell wall, starch, sucrose, amino acid, lipid and protein degradation, thus providing alternative sources of energy and metabolites. Furthermore, SnRK1 represses a set of energy-consuming ribosome biogenesis and anabolism genes and also slows down the growth of roots and shoots (Baena-González *et al.* 2007). A second contribution to the reduction in growth rate in submerged *Ru. acetosa* appears to come from enhanced abscisic acid (ABA) signalling. This is indicated by the lack of a submergence-induced ABA decline and enhanced expression of downstream ABA signalling components (van Veen *et al.* 2013). Previous studies have shown that a decline in endogenous ABA levels in *Ru. palustris* petioles is a prerequisite for underwater elongation growth (Benschop *et al.* 2005) since ABA represses transcription of gibberellin (GA) biosynthesis genes (Benschop *et al.* 2006). The picture emerges that *Ru. acetosa* is able to reprogramme metabolism and hormone homeostasis towards catabolism and growth inhibition. Interestingly, this flooding survival strategy is initiated without substantial shortages of O_2_, carbohydrates and ATP, suggesting an anticipation of these future events. It is tempting to speculate that ethylene drives these metabolic and growth modifications.

The escape strategy employed by *Ru. palustris* depletes starch and sucrose probably to fuel fast elongation growth (van Veen *et al.* 2013). This is consistent with earlier findings that underwater elongation was hampered in carbohydrate-starved plants (Groeneveld and Voesenek 2003). The underwater elongation response is initiated by accumulation of ethylene and subsequently a decline in ABA and an increased biosynthesis of and sensitivity towards GA. Important target processes required for cell elongation are apoplastic acidification and cell wall loosening (Vreeburg *et al.* 2005; Bailey-Serres and Voesenek 2008; van Veen *et al.* 2013). Recently, an additional pathway was added to the molecular cascade that regulates fast petiole elongation under water in *Ru. palustris*. This species uses gene products associated with photomorphogenesis and shade avoidance (*COP1*, *KIDARI*, *HD-ZIPII*, *PIF*) for submergence-induced elongation. This light signalling pathway is activated by ethylene and ABA and operates independently of the phytochromes (van Veen *et al.* 2013). This ethylene-driven activation of light signalling genes during flooding-induced shoot elongation is not observed in elongating rice varieties (Jung *et al.* 2010). This suggests that fast shoot growth in response to shade and submergence cues are not only phenotypically very similar (Pierik *et al.* 2010), but that they also share a conserved downstream signalling pathway.

As mentioned earlier, if these escaping plants cannot emerge out of the flood water, their survival is compromised (Setter and Laureles 1996). Surprisingly, our survival data for plants submerged in darkness indicate similar LT_50_ values (20 days) for both *Rumex* species despite their contrasting elongation strategies (van Veen *et al.* 2013). Interestingly, *Ru. palustris* showed a stronger upregulation of the so-called core-hypoxia genes (Mustroph *et al.* 2009, 2010), a hallmark of low-O_2_ adaptation in a broad range of species, despite the absence of a marked depletion of O_2_ in its submerged shoots (van Veen *et al.* 2013). Some of these core-hypoxia genes are directly upregulated by elevated levels of ethylene. Additionally, many of the core-hypoxia genes in *Ru. palustris* can be upregulated experimentally by anoxia and the effect is enhanced by an ethylene pretreatment. These observations suggest that ethylene can prime *Ru. palustris* for future anoxia tolerance. Indeed, plants pretreated with ethylene showed higher survival and more vigorous recovery after an anoxia period (van Veen *et al.* 2013). This mechanism might explain the relatively long survival under submerged dark conditions of *Ru. palustris* despite expending resources on its strong elongation response. Remarkably, this priming mechanism was absent in *Ru. acetosa* (van Veen *et al.* 2013).

As mentioned before, in Arabidopsis, group VII ERF transcription factors are involved in O_2_ sensing. In the presence of O_2_, members of this transcription factor family are targeted for degradation via the N-end rule pathway of protein degradation (Gibbs *et al.* 2011; Licausi *et al.* 2011). Interestingly, mutants (*ate1ate2* and *prt6*) that impair the N-end rule pathway of targeted proteolysis and stabilize the ERFs show constitutive expression of many of the core-hypoxia genes, indicating that these stable ERFs regulate the expression of genes important for surviving anaerobiosis (Gibbs *et al.* 2011). These observations feed the hypothesis that ethylene-regulated anoxia priming might work via increased stability of group VII ERFs and therefore enhanced expression of downstream adaptive anaerobic genes during ethylene exposure and even more during subsequent anoxia. This novel role of the volatile hormone ethylene is consistent with the sequence of events that take place during natural flooding events. The rate of O_2_ decline is often gradual and the rate unpredictable as tissues progress from hypoxia to anoxia over hours or days. In contrast, ethylene accumulates throughout the plant within minutes of submergence, making it a more reliable and timely (early) signal of submergence (Voesenek and Sasidharan 2013).

## Perspectives/Concluding Remarks

Studies in *Rumex* and *Rorippa* have delivered unprecedented insights into flooding tolerance mechanisms and demonstrate the value of examining wild plant species. From these and earlier performed investigations, it becomes clear that the long-term survival of submergence can only be achieved with a balanced control of energy-demanding and energy-producing processes. In this, activation of catabolism, reduction of elongation growth, and controlled glycolysis and ethanolic fermentation to increase substrate-level production of ATP are key processes. This level of control might also determine variation in waterlogging tolerance in species that cannot avoid low O_2_ conditions due to very low root porosities.

The unbiased study into the regulation of enhanced shoot elongation in submerged *Ru. palustris* revealed that signalling genes well known in photomorphogenesis and shade avoidance responses are regulated by accumulated ethylene and reduced levels of ABA and are therefore likely to play a role in ethylene-driven shoot elongation. We conclude that *Ru. palustris* uses a conserved signal transduction network that regulates elongation growth to both submergence and changes in the light spectrum (e.g. shade avoidance). This explains the high phenotypic similarity of shade avoidance responses and submergence-stimulated elongation (Pierik *et al.* 2010).

The observed upregulation of core-hypoxia genes in well-aerated submerged petioles of *Ru. palustris* led to the discovery of a novel ethylene-induced priming mechanism of anoxia tolerance. This mechanism was entirely absent in *Ru. acetosa*, indicating that priming by ethylene is not a general response of plants to flooding but linked to enhanced tolerance. This ‘ethylene training’ resembles the observation that hypoxia-pretreated roots are more anoxia tolerant, have more alcohol dehydrogenase (ADH) activity and maintain higher levels of ATP (Saglio *et al.* 1988). The significant increase in recovery and survival of plants pretreated with ethylene has high potential to improve the flooding tolerance of agronomically important crops. The next steps will be to identify the underlying regulators.

Improved waterlogging and submergence tolerance of crops is important because more frequent and prolonged flooding events will increasingly hamper crop productivity in the future as a direct consequence of climate change (Kundzewicz *et al.* 2013). A dramatic example comes from South China where 30 % of the seeds for worldwide rapeseed oil production are grown as a rotation crop in rice paddy fields (Zhang *et al.* 2008). Young seedlings of this flooding-intolerant plant are frequently exposed to waterlogging, causing an annual yield loss of ∼17–42 % (Zhou and Lin 1995). Furthermore, the world human population is expected to increase from the current 7 billion to ∼9 billion by 2050, requiring us to increase current agricultural productivity by a similar or greater proportion if widespread hunger and social unrest are to be averted (Tester and Langridge 2010). Elucidation of the molecular mechanisms that regulate flooding tolerance in model plants such as rice and Arabidopsis is important, but this approach should be accompanied by investigations into as yet unknown flood-adaptive traits in wild plant species that have evolved to cope with flooding stress. The *Rorippa* and *Rumex* studies demonstrate that whole-genome transcription profiling technologies can be successfully applied to non-model species to uncover novel tolerance genes and processes.

Important future challenges are the development of crops that can re-allocate their energy and carbon reserves in response to early flooding signals to support maintenance rather than growth and other costly processes. Most flooding damage to crops is related to partial flooding or waterlogging of the soil. Most waterlogging-tolerant wild species have an extensive system of interconnected gas spaces (aerenchyma) that is either stress inducible or constitutive (Colmer 2003). Aerenchyma development is a complex trait. It involves lysis of specific cells or schizogeny during root development (Voesenek *et al.* 2006). Understanding the molecular background of aerenchyma formation will facilitate the development of crops rich in root aerenchyma. Important in this respect is the observed trade-off between root cortical aerenchyma formation and nutrient transport in *Zea mays* (Hu *et al.* 2013). This could potentially hamper the productivity of these aerenchyma-rich varieties. A final challenge is exploring the potential of ethylene-induced priming for anoxia tolerance. Identification of the molecular regulators of ethylene-driven priming can be expected to lead to the introduction of this adaptive trait in crops that currently lack it.

## Sources of Funding

H.v.V. and R.S. have been financially supported by the Netherlands Organisation for Scientific Research (grants 819.01.006 to H.v.V. and VENI 86312013 to R.S.).

## Contributions by the Authors

L.A.C.J.V. and R.S. wrote the paper; R.S. and H.v.V. performed many of the *Rumex* and *Rorippa* experiments and analyses.

## Conflicts of Interest Statement

None declared.
